# Characterization of a Small Supernumerary Marker Chromosome Derived from Xq28 and 14q11.2 Detected Prenatally

**DOI:** 10.1155/2018/2875241

**Published:** 2018-05-07

**Authors:** Akihiro Hasegawa, Osamu Samura, Taisuke Sato, Tomona Matsuoka, Yuki Ito, Kazuhiro Kajiwara, Hiroaki Aoki, Yuka Inage, Masahisa Kobayashi, Aikou Okamoto

**Affiliations:** ^1^Department of Obstetrics and Gynecology, The Jikei University School of Medicine, 3-25-8 Nishi-Shinbashi, Minato-ku, Tokyo 105-8461, Japan; ^2^Department of Pediatrics, The Jikei University School of Medicine, 3-25-8 Nishi-Shinbashi, Minato-ku, Tokyo 105–8461, Japan

## Abstract

We present the characterization of a case with a small supernumerary marker chromosome (sSMC) detected prenatally derived from Xq28 and 14q11.2 maternal translocation. A 33-year-old Japanese woman, primigravida, underwent amniocentesis because of fetal growth restriction and fetal structural abnormality at 30 weeks of gestation. The fetal karyotype was identified as 47,XY,+mar. Additionally, the single nucleotide polymorphism array analysis revealed copy number gains at Xq28 and 14q11.2. A male infant, weighing 1,391 g, was delivered at term by cesarean section. Maternal and paternal karyotypes were 46,X,t(X; 14)(q28; q11) and 46,XY, respectively. These findings indicated that the sSMC might have originated from chromosome disjunction at a ratio of three to one. Here we describe a case with an sSMC derived from Xq28 and 14q11.2. Our findings suggest that this sSMC is most likely pathogenic. The collection of additional cases may be required.

## 1. Introduction

A small supernumerary marker chromosome (sSMC) is defined as an abnormal chromosome that is generally equal in size to or smaller than chromosome 20, and its derivation cannot be detected by conventional chromosomal banding techniques [1]. These chromosomes are detected in 0.08% of unselected prenatal cases and in 0.20% of prenatal cases with a fetal abnormality by ultrasonography [2]. The phenotypes resulting from sSMC duplication vary widely depending on the origin. Phenotypes have been normal in several cases in which an sSMC was detected, and the effects of sSMCs on the phenotype remain unclear.

We experienced a case with an sSMC derived from Xq28 and 14q11.2. As noted in previous reports, the most notable gene in the Xq28 region is the methyl-CpG binding protein 2 gene* (MECP2)* [3]. Duplication of* MECP2* results in severe intellectual disability and dysphasia, seizure, and recurrent infectious disease [3]. However, to the best of our knowledge, there has been only one previous report on 14q11.2 duplication with short stature and mild intellectual disorder, hypogenitalism, and retrognathia [4]. We present herein the characterization of a case with an sSMC derived from Xq28 and 14q11.2 detected prenatally.

## 2. Case Presentation

**Figure 1:**
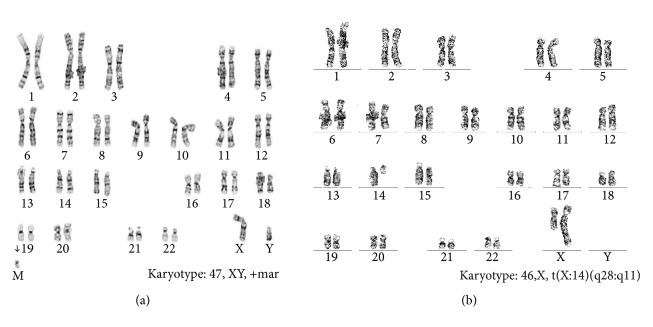
Results of fetal and maternal karyotype: (a) fetal karyotype determined from amniotic fluid cells at 31 weeks of gestation and (b) maternal karyotype detected from lymphocytes of maternal blood.

**Figure 2:**
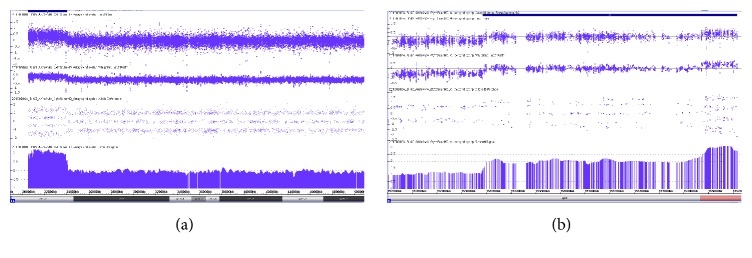
Results of the SNP microarray analysis: (a) karyotype view of the 14q11.2 duplication and (b) karyotype view of the Xq28 duplication.

**Figure 3:**
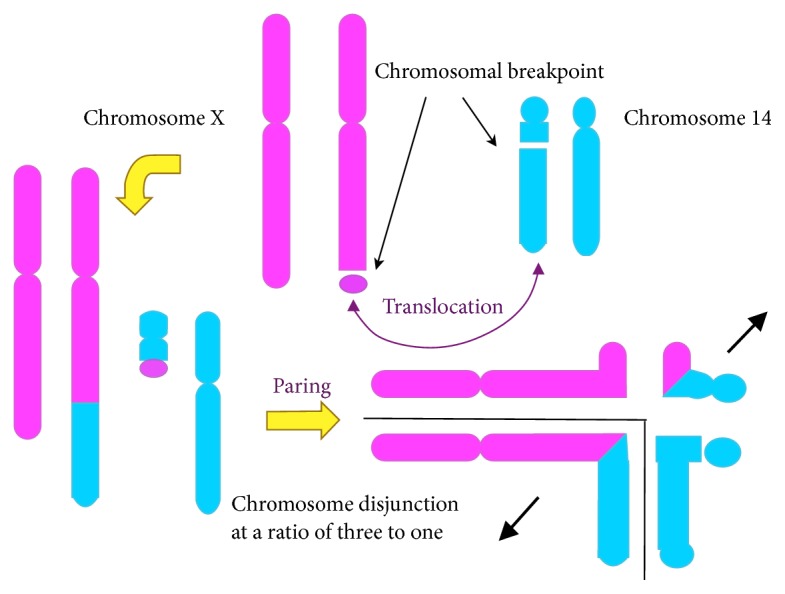
Pachytene scheme of fetal chromosomal abnormality.

A healthy 33-year-old Japanese woman, gravida one, para zero, with no family history of malformations or genetic disorders, was referred to our hospital because of fetal growth restriction and multiple fetal malformations at 30 weeks of gestation. Ultrasonography findings revealed severe fetal growth restriction and micrognathia and overlapping fingers. We also suspected esophageal atresia because of polyhydramnios and no detection of gastric pouch. The fetal karyotype was identified as 47,XY,+mar by amniocentesis at 31 weeks of gestation (Figure 1(a)). We suspected sSMC derived from the maternal balanced translocation and performed an additional SNP microarray analysis of amniotic fluid cells. The SNP microarray revealed a 3.55 MB proximal duplication of 14q11.2 and a 2.10 MB terminal duplication of Xq28 as well as a pseudoautosomal region (Figure 2). We provided adequate genetic counseling to inform the patient and her husband about the prognosis according to our best estimates. A live male infant weighing 1,391 g was delivered at 38 weeks of gestation by emergency cesarean section, because the cardiotocogram after the rupture of the membrane showed a nonreassuring fetal status with severe prolonged deceleration and mild late deceleration with decreasing variability. The Apgar scores at one minute and five minutes were four points and eight points, respectively. The physical parameters were as follows: the height was 37.0 cm, head circumference was 31.6 cm, and thoracic circumference was 25.0 cm. The umbilical artery pH was 7.253. The infant exhibited overlapping fingers, micrognathia, contracture of the lower extremities, and severe dysphasia resulting in polyhydramnios despite a lack of esophageal atresia. Cytogenetic studies were performed on the patients using standard techniques. The results of the maternal and paternal karyotypes were 46,X,t(X;14)(q28;q11) and 46,XY, respectively (Figure 1(b)). Therefore, we concluded that the sSMC might have originated from chromosome disjunction at a ratio of three to one (Figure 3). The infant exhibited dysphasia and deafness. Severe hypogammaglobulinemia that required replacement therapies was also detected. Additionally, chest computed tomography showed stenosis of the right intermediate bronchus. The infant underwent tracheostomy to increase positive airway pressure and stabilize respiration. As his respiratory condition stabilized, the infant also underwent an orchiopexy for right cryptorchidism at 8 months after birth. Magnetic resonance imaging (MRI) findings at 9 months after birth revealed cerebral ventriculomegaly, cerebral atrophy, and subependymal hemorrhage. The infant had delayed head control and difficulties with eye contact; therefore, he was diagnosed with severe intellectual disability. At 340 days after birth, he was discharged from our hospital with a respirator.

## 3. Discussion

We present the characterization of a case with an sSMC derived from Xq28 and 14q11.2 maternal translocation detected prenatally. To the best of our knowledge, there have been no previous reports of a case with an sSMC derived from Xq28 and 14q11.2 maternal translocation.

The most notable gene in the Xq28 region is the* MECP2* gene. Most cases of* MECP2* duplication syndrome occur in males, and almost 50% of these patients die before 25 years of age [5]. The duplication of* MECP2* results in severe intellectual disability and dysphasia, seizure, and recurrent infectious disease [3]. According to the findings of genetic testing used for diagnosis, duplications of* MECP2* ranging from 0.3 to 4 Mb and larger have been detected in all cases. Previous reports noted the following symptoms at clinical diagnosis: (i) severe intellectual disability, with absent or limited speech; (ii) early-onset hypotonia with slow motor development; (iii) progressive spasticity predominantly of the lower limbs; (iv) predisposition to recurrent respiratory infections; (v) epilepsy, and (vi) other variably present characteristics, including autistic features, gastrointestinal dysfunction, and mild facial dysmorphism [5].

However, to the best of our knowledge, there have been few previous reports on 14q11.2 duplication. Duplication of 14q11.2 is characterized by a short stature and mild intellectual disability, hypogenitalism. A birth record showed the following signs or conditions: birth weight 2,350 g (<3rd centile), length 45 cm (<3rd centile), iris coloboma at the left eye, and retrognathia. A patient started to walk at 16 months and talk for the first time at 2-years-old. The previous report showed a few candidate genes in the duplicated region that might be involved in the intellectual disability, such as* CPNE6*,* NEDD8*, and* LOC401744*. One previous report showed that 14q11.2 duplication could affect the phenotype [4]. In addition, one case of similar duplication was reported. The patient with a cryptic unbalanced translocation t(14; 15)(q11.1; q11.2) causing monosomy for 15q11 and trisomy for 14q11 presented with an unusual Angelman syndrome with an extremely short stature and severe intellectual disability, lack of speech, and seizure ataxic gait [6]. In this case, copy number gain of Xq28 and 14q11.2 was detected prenatally based on the results of an SNP microarray for an sSMC. As noted above, intellectual disability is the chief symptom of* MECP2* syndrome without the structural abnormalities. The structural abnormalities including micrognathia might have been caused by the 14q11.2 copy number gain. The recurrent risk of the same chromosomal abnormality is difficult to identify because it depends on the type of disjunction and X-inactivation [7].

In conclusion, we present the characterization of the first case with an sSMC detected prenatally, derived from Xq28 and 14q11.2 maternal translocation. Our findings suggest that this sSMC is most likely pathogenic. A definitive determination may require the collection of additional cases.
